# Opioid prescription patterns in long-term disease-free cancer survivors: a retrospective analysis at a single NCI-designated comprehensive cancer center

**DOI:** 10.1007/s00520-025-10003-z

**Published:** 2025-11-03

**Authors:** Vasco M. Pontinha, Livingstone Aduse-Poku, Gerard F. Moeller, Renato G. Martins, Danielle Noreika, Susan Hong

**Affiliations:** 1 Department of Pharmacotherapy and Outcomes Science, VCU School of Pharmacy, Richmond, VA USA; 2 VCU Massey Comprehensive Cancer Center, Virginia Commonwealth University, Richmond, VA USA; 3 Department of Psychiatry, VCU School of Medicine, Richmond, VA USA; 4 Division of Hematology, Oncology, and Palliative Care, Department of Medicine, VCU School of Medicine, Richmond, VA USA; 5 Division of Hematology/Oncology, Department of Internal Medicine, VCU School of Medicine, Richmond, VA USA

**Keywords:** Opioid, Cancer survivors, Pain management, Narcotics

## Abstract

**Purpose:**

This study examined opioid dose trajectory patterns in a cohort of disease-free cancer survivors who were at least 1 year out from completion of their cancer treatment at a single NCI-designated comprehensive cancer center.

**Patients and methods:**

We conducted a retrospective observational cohort study using electronic health records. Individuals diagnosed and treated for cancer with opioid prescriptions between 2004 and 2024 were identified through a combined review of ICD-10/ICD-9 codes. Inclusion criteria encompassed disease-free cancer patients who were prescribed opioids at least 1 year after completion of cancer treatment. Patients with cancer and sickle cell disease or receiving palliative care were excluded from the analysis. Prescriptions were standardized to average daily morphine milligram equivalent (MME) for a period of 24 months. Participants were classified as high-dose (≥ 50 MME/day) or low-dose (< 50 MME/day) persisters based on the strength of their average daily MMEs 1 year after completion of their cancer treatment. Prescription patterns were elicited using group-based trajectory modeling, and linear mixed-effects regression models.

**Results:**

A total of 1688 disease-free cancer survivors were identified, with 610 being prescribed opioids 1 year after completion of their cancer treatment. Low-dose persisters (*n* = 404) exhibited two trajectories: discontinuers (61.7%) and escalators (38.3%). Low-dose escalators increased from < 50 MMEs/day to an average of 100 MMEs/day at 24 months. High-dose persisters, i.e., individuals on ≥ 50 MMEs/day (*n* = 206) at 1 year after completion of their cancer treatment, exhibited escalating doses up to 250 MMEs at 24 months. Sex and race were the only sociodemographic characteristics found to be significant predictors of continued opioid exposure.

**Conclusion:**

In our cohort of 610 disease-free cancer survivors on opioids at least 1 year after completion of their cancer treatment, 59.2% (*n* = 361) were prescribed escalating doses of opioids. Since higher opioid doses have been shown to be associated with increased risks of harm, future multicenter studies are needed to examine the factors associated with increasing opioid doses as well as intervention strategies to mitigate opioid escalation in disease-free cancer survivors.

## Introduction

As of January 1, 2025, there are about 18.6 million cancer survivors (CS), and by 2035, it is expected that over 22 million Americans will be considered cancer survivors [1]. Despite the progress made in cancer care, many cancer survivors face lasting treatment-related complications, including chronic pain. For example, Jian and colleagues demonstrated that 36.4% of patients with a history of cancer reported chronic pain [2]. Furthermore, of those with chronic pain, an estimated 5 to 10% reported pain severe enough to interfere with functioning [3].

During active cancer treatment, pain is often the result of ongoing tissue damage either due to the malignancy itself or from cancer treatments. At this time, opioids can be an effective tool in managing moderate to severe pain. However, many CS continue on opioid therapy years after completion of their primary cancer treatment when tissue injury is no longer occurring [4]. This is problematic, as there is little evidence to support the use of opioids in chronic pain management [5]. In addition, according to the American Society of Clinical Oncology (ASCO), research examining the overall benefits of opioid use in long-term CS is strikingly small [6].

In the non-cancer population, continued exposure is linked to opioid-related harms, including the development of opioid-use disorder (OUD), overdose, and death [5, 7]. Long-term opioid use is also associated with increased healthcare utilization due to opioid-related hospitalizations or emergency department (ED) visits [8]. In the cancer population, there is a growing body of preclinical and observational studies on the harms of opioid use indicating a potential association with risk of malignancy recurrence and poorer cancer survival [6, 9–18].

Although numerous studies have explored the rates of persistent opioid use in CS after completion of treatment, variations in how persistent opioid use is defined complicate the estimation of the true prevalence of chronic opioid use in disease-free cancer survivors. For example, in a population-level analysis of 38,366 CS in North Carolina, persistent opioid use was defined as at least 90 days of prescribed opioids between 1 and 2 years after diagnosis. In this cohort, only 3% were considered persistent opioid users [19]. In another study of 106,732 military veteran CS diagnosed between 2000 and 2015, Vitzthum and colleagues found the rate of persistent opioid use, defined as filling 120 days or more of opioid prescriptions 1–2 years after the start of curative treatment, to be 8.3% [4]. Other studies have reported estimates of persistent opioid use ranging between 10.4 [20] and 33.3% [21]. Since duration of opioid use is linked to increased risk of harms, it is important to understand the true prevalence of persistent opioid use in the CS population [22].

To date, no studies have examined the overall dose of opioids prescribed over time in CS. Instead, studies typically characterize opioid exposure dichotomously, i.e., still using opioids or not [4, 23–25]. Other studies investigated opioid exposure during the perioperative stages of treatment with curative intent, instead of a well-defined period in which patients are not undergoing any type of cancer treatment [22, 24, 26–29]. Since dose of opioids is also linked to increased risk of harms, information on dose patterns during cancer survivorship remains a significant literature gap and is needed to fully understand the potential health impacts of opioids in disease-free cancer survivors [22, 26–30].

In order to fill this gap, our study sought to characterize the patterns of opioid prescriptions in a population of disease-free cancer survivors who were at least 1 year out of active cancer treatment at a single NCI-designated comprehensive cancer center. All patients were served by the Virginia Commonwealth University Massey Comprehensive Cancer Center (VCU Massey).

## Methods

### Study design

**Fig. 1:**
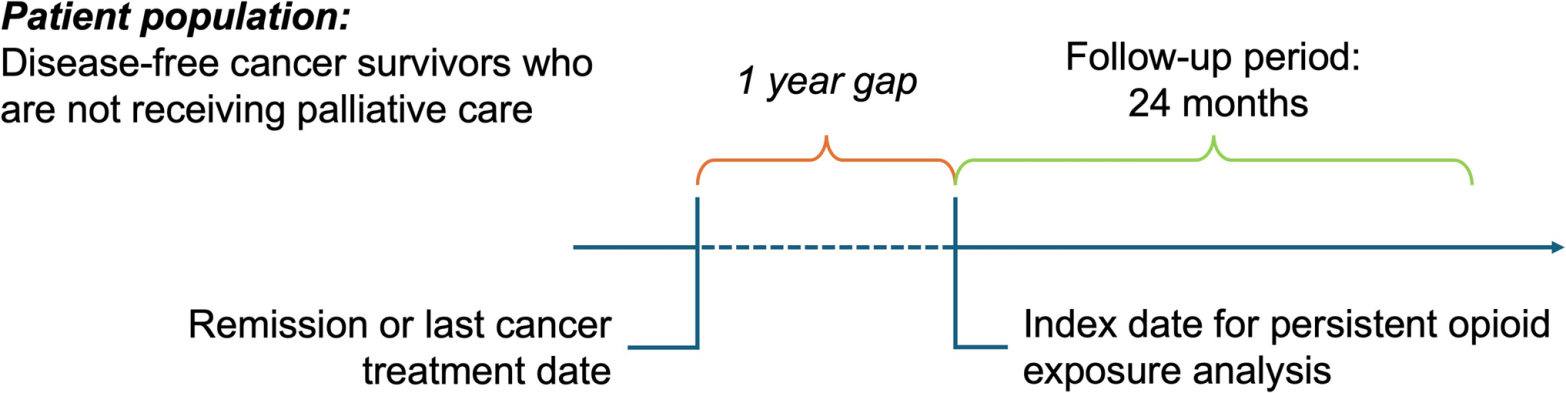
Study design schema

We conducted a retrospective longitudinal observational study by querying the electronic health records (EHR) of patients with a cancer diagnosis at VCU Massey to characterize the prescription patterns of opioids used in the outpatient setting. Study participants were identified through ICD-10 or ICD-9 codes for a single primary cancer diagnosis between January 2004 and June 2024. Patients with multiple primary diagnoses were excluded from the analysis. Participants were included in the study if they 1) were disease-free, 2) were still being prescribed opioids at least 1 year after the date of completion of their cancer treatment or remission, and 3) received care at VCU Massey for an additional period of 24 months (Fig. 1). Disease-free status was identified via EHR review based on clinical documentation. Patients who were disease-free on oral adjuvant agents designed to decrease the risk of cancer recurrence, such as breast cancer patients on aromatase inhibitors, were included in the analysis. Sociodemographic characteristics such as race, ethnicity, insurance status, and zip code were informed by the EHR. Social deprivation scores, overall and health-related, were calculated based on the zip code according to Robert Graham Center SDI Scores [31]. Participants were excluded if they were receiving palliative care (as these patients likely had a recurrence during the follow-up period). This was informed by the respective ICD-10 or ICD-9 diagnosis codes, Z51.5 or V66.7. Participants were also excluded if they were taking opioids via any route other than oral or transdermal, as they were considered to be inpatient exclusive. In addition, cancer patients with sickle cell disease were excluded from the analysis, given their ongoing need for opioids to manage their vaso-occlusive episodes [32, 33]. Prescription orders were queried from the EHR and standardized to average daily morphine milligram equivalent (MME) using the CDC Opioid Oral Morphine MME Conversion Factors [34]. Only complete prescription records were included in the analysis. Prescriptions were excluded from the analysis when information on dosage or quantity supplied was missing. Records were also excluded if dosage information was described as “as needed” (i.e., PRN), as it was not possible to calculate the average daily MME given that either frequency of daily use or overall quantity dispensed was often missing. The remaining records in the analysis were grouped by the strength of the first opioid prescribed at least 1 year after completion of their last active cancer treatment (specifically chemotherapy, radiation, immunotherapy, or surgery), following the 2022 CDC opioid prescribing guideline and accounting for the risk of escalating doses of opioids. Therefore, participants whose average daily dose at the index date was 50 MME/day or higher were considered “HIGH-DOSE PERSISTERS,” whereas those under 50 MME/day were considered “LOW-DOSE PERSISTERS” [5]. This study was approved by the Virginia Commonwealth University Institutional Review Board (IRB) in compliance with the Declaration of Helsinki.

### Statistical analysis

Models for 2–5 trajectories were estimated using group-based trajectory modeling (GBTM). GBTM clusters participants with similar distinctive features according to their prescribed average daily dose in MME [35]. The Bayesian information criterion (BIC) was estimated to identify the model with the best-fitting number of trajectories [36, 37]. The best-fitting model selection was the model with the BIC closest to 0 [35, 37]. A Bayesian approach was also implemented to examine model adequacy by estimating the absolute model fit statistics [38]. The Bayesian parameters that were estimated included average posterior probabilities of each trajectory, odds of correct classification, and observed classification proportion versus the expected classification. Good fit was indicated when each trajectory in the model yielded an average posterior probability > 70% and the odds of correct classification were equal to 5 or higher [36, 39]. The shape of each trajectory was determined by specifying the better-fitting polynomial function. The highest order indicating a *p*-value over 0.05 was considered the better-fitting polynomial function [35, 37, 39]. When group-based trajectory models were not able to be computed due to small sample size (*n* = ≤ 300) or dispersed prescription data, linear mixed-effects regression (LME) models were used instead. This alternative approach is useful for unbalanced data, with a differing number of observations per individual over time and permits the inclusion of time-variant and -invariant predictors of the population-level trend [40, 41]. Ultimately, absent the ability to determine different opioid prescription trajectories, LME models would allow for determining the evolution of the average daily opioid dose prescribed considering random within-subject variation.

### Predictors of continued opioid use during cancer survivorship

The adjusted risk model accounted for characteristics including type of cancer (i.e., site of primary cancer diagnosis), exposure to opioids prior to cancer diagnosis, Hispanic ethnicity, sex, race, and residence in a rural or urban area. Statistical significance was examined following a stepwise approach.

The group-based trajectory analyses and alternative regression models were computed using STATA 16 IC, including the STATA plugin *traj*, assuming a significance level *α* = 0.05 [42, 43].

## Results

From an initial sample of 21,825 patients with a history of malignancy, 1,688 disease-free cancer survivors 1 year after the date of last treatment were identified. Of these, 610 (36.1%) disease-free cancer survivors were still being prescribed an opioid at least 1 year after their last cancer treatment, with 404 (66.2%) classified as low-dose persisters and 206 (33.8%) as high-dose persisters (Table 1). Average age at diagnosis was slightly.

**Table 1:** Sample characteristics

	1 year disease-free cancer survivors exposed to opioids	Low-dose persisters	High-dose persisters	∆ test
*N*	610 (100.0%)	404 (66.2%)	206 (33.8%)	
Age at diagnosis	56.5 (12.0)	57.3 (12.4)	54.8 (11.1)	0.015
Sex				0.732
Male	278 (45.6%)	182 (45.0%)	96 (46.6%)	
Female	331 (54.3%)	221 (54.7%)	110 (53.4%)	
Unknown	1 (0.2%)	1 (0.2%)	0 (0.0%)	
Race				< 0.001
White	257 (44.9%)	152 (41.4%)	105 (51.0%)	
Black	287 (50.1%)	210 (57.2%)	77 (37.4%)	
Asian	1 (0.2%)	0 (0.0%)	1 (0.5%)	
Two or more races	7 (1.2%)	5 (1.4%)	2 (1.0%)	
Unknown	21 (3.7%)	0 (0.0%)	21 (10.2%)	
Ethnicity				0.646
Not Hispanic^a^	599 (98.2%)	396 (98.0%)	203 (98.5%)	
Hispanic	11 (1.8%)	8 (2.0%)	3 (1.5%)	
Unknown				
Area of residence				0.176
Urban metro area	546 (89.5%)	367 (90.8%)	179 (86.9%)	
Small town	31 (5.1%)	20 (5.0%)	11 (5.3%)	
Rural area	33 (5.4%)	17 (4.2%)	16 (7.8%)	
Social deprivation^b^				
Overall score	0.4 (0.5)	0.4 (0.5)	0.3 (0.5)	0.034
Healthcare score	0.1 (0.3)	0.1 (0.2)	0.1 (0.3)	0.121
Type of 1 st cancer				0.003
Head and neck	61 (10.2%)	35 (9.0%)	26 (12.6%)	
Lung	91 (15.2%)	65 (16.6%)	26 (12.6%)	
Pancreas	7 (1.2%)	5 (1.3%)	2 (1.0%)	
Gastrointestinal	29 (4.9%)	19 (4.9%)	10 (4.9%)	
Gynecologic cancers and breast^c^	122 (20.4%)	88 (22.5%)	34 (16.5%)	
Prostate	35 (5.9%)	29 (7.4%)	6 (2.9%)	
Leukemia/lymphoma	118 (19.8%)	58 (14.8%)	60 (29.1%)	
Kidney and Urinary	33 (5.5%)	23 (5.9%)	10 (4.9%)	
Brain	13 (2.2%)	7 (1.8%)	6 (2.9%)	
Kaposi sarcoma	1 (0.2%)	0 (0.0%)	1 (0.5%)	
Colon/rectum	53 (8.9%)	40 (10.2%)	13 (6.3%)	
Musculoskeletal/heart	17 (2.8%)	10 (2.6%)	7 (3.4%)	
Skin/Melanoma	10 (1.7%)	8 (2.0%)	2 (1.0%)	
Unknown	7 (1.2%)	4 (1.0%)	3 (1.5%)	
Opioid exposure prior to cancer				< 0.001
Previously exposed	322 (52.8%)	241 (59.7%)	81 (39.3%)	
Opioid-naïve	288 (47.2%)	163 (40.3%)	125 (60.7%)	
Insurance status				0.155
Commercial Insurance	179 (29.3%)	122 (30.2%)	57 (27.7%)	
TRICARE	3 (0.5%)	1 (0.2%)	2 (1.0%)	
Medicare	159 (26.1%)	111 (27.5%)	48 (23.3%)	
Medicaid	108 (17.7%)	62 (15.3%)	46 (22.3%)	
Dual-eligible	46 (7.5%)	33 (8.2%)	13 (6.3%)	
Not insured	111 (18.2%)	71 (17.6%)	40 (19.4%)	
Unknown	4 (0.7%)	4 (1.0%)	0 (0.0%)	

^a^Hispanic: Self-identified as Hispanic, Latino(a), or of Spanish origin. ^b^The Social Deprivation Index [20] is a composite measure of area-level deprivation based on seven domains retrieved from the American Community Survey (ACS) used to quantify socioeconomic variation in health outcomes: i) proportion of people under the federal poverty limit, ii) level of education, iii) employment, iv) homeownership, v) population density, vi) single-families with dependents, and vii) vehicle ownership. ^C^No male was diagnosed with breast cancer

higher for low-dose persisters (57.3 years) compared to high-dose persisters (54.8 years), with a significant difference (*p* = 0.015). Sex distribution was similar between the groups, with males comprising 45.0% of low-dose persisters and 46.6% of high-dose persisters. Racial composition showed a significant difference (*p* < 0.001), with a higher percentage of Black participants in low-dose persisters (57.2%) compared to high-dose persisters (37.4%). Most participants identified as non-Hispanic (98.2%), and the majority lived in urban metro areas (89.5%). Social deprivation scores were slightly higher in low-dose persisters (0.4) compared to high-dose persisters (0.3). The type of first cancer varied significantly (*p* = 0.003) across groups, with a higher prevalence of leukemia/lymphoma in high-dose persisters (29.1%) compared to low-dose persisters (14.8%). Opioid exposure prior to cancer diagnosis was significantly different (*p* < 0.001), with 59.7% of low-dose persisters having previous exposure compared to 39.3% of high-dose persisters.

### Low-dose persisters

**Fig. 2:**
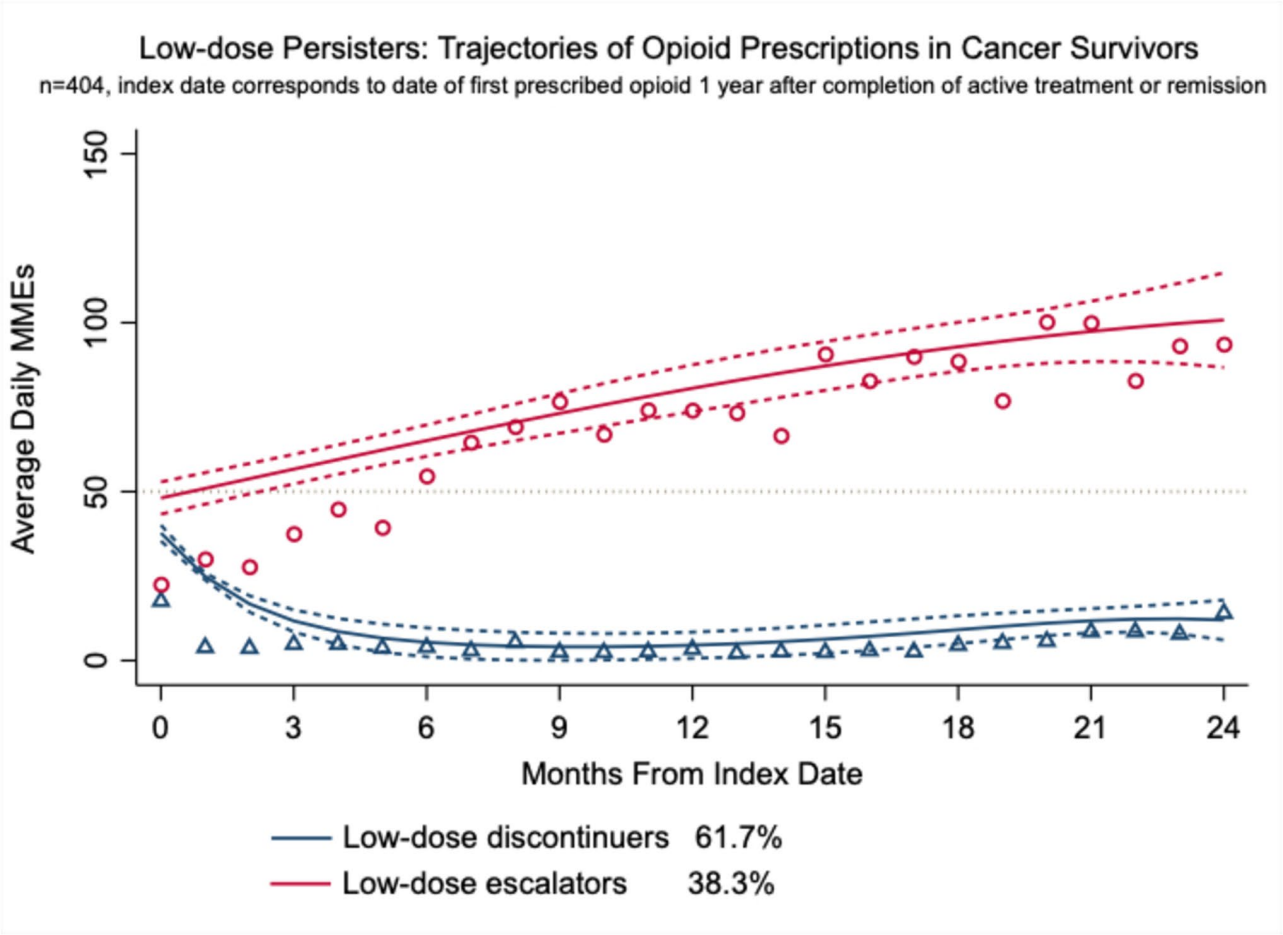
Trajectories of low-dose persistent users of opioid during cancer survivorship

**Fig. 3:**
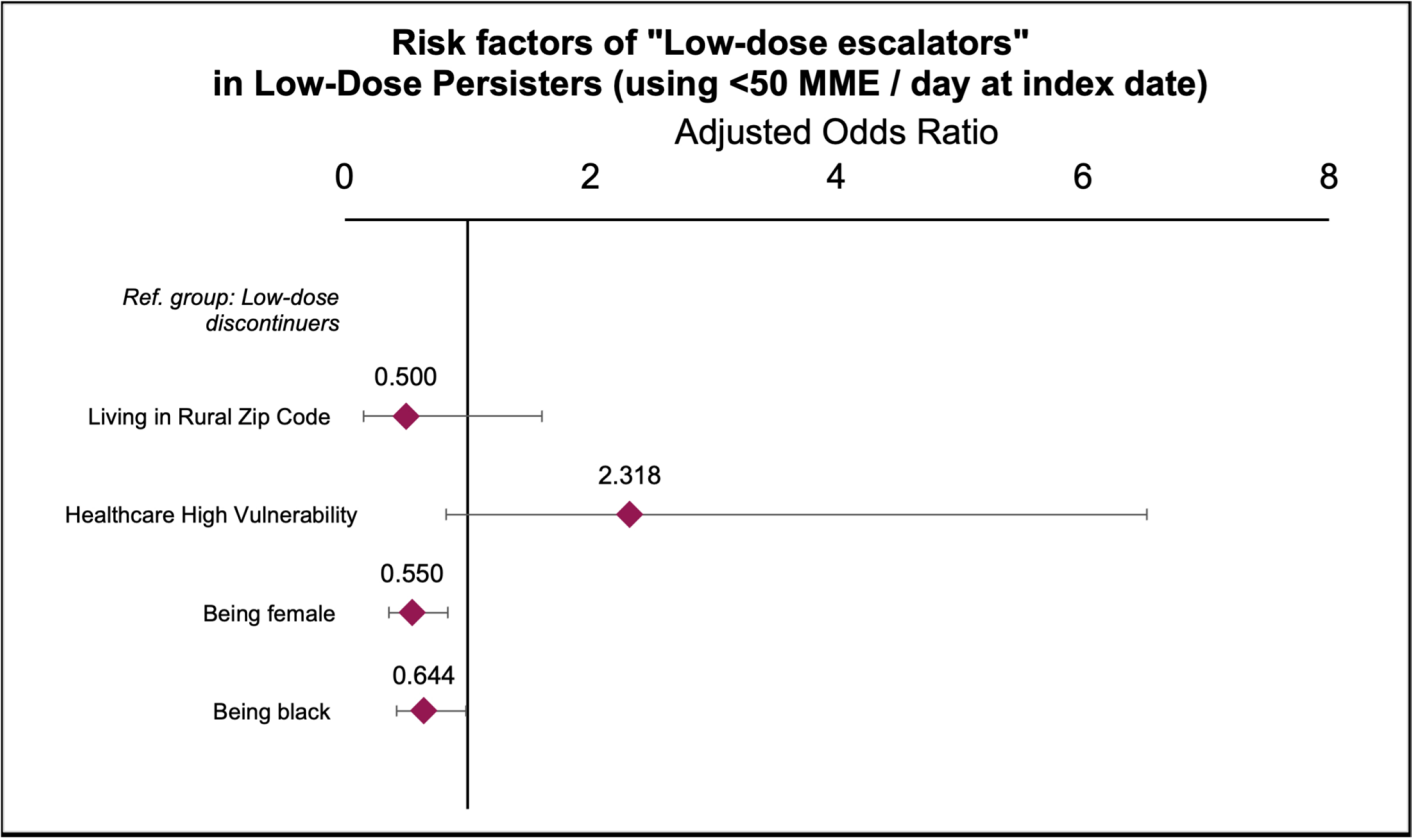
Predicting risk factors of following low-dose escalators trajectory compared to low-dose discontinuers

Prescription data from low-dose persisters exhibited two distinct trajectories. The first, the “low-dose discontinuers,” showed a progressive decrease in the average daily dose of opioids trending to zero. The second, the “low-dose escalators” trajectory, demonstrated a continued increase in the average daily dose, reaching approximately 100 MMEs/day (Fig. 2). An adjusted risk model was implemented to examine potential predictors associated with the persistent use trajectory. Following a stepwise approach, living in a rural zip code, being female, or Black were found to be protective factors against low-dose discontinuers (Fig. 3). Nevertheless, rurality lost statistical significance in the final adjusted model. The same was found for living in a zip code with high healthcare deprivation [31]. Of note, our models show that 37.8% of low-dose escalators who started with doses less than 50 MME/day progressed to an average dose of approximately 100 MMEs/day within 24 months. All trajectories in the model exhibited average posterior probabilities higher than 70% and odds of correct classification higher than 5, suggesting good model fit. Table 2 contains the absolute fit statistics of the trajectory model.

**Table 2:** Absolute group-based trajectory model fit statistics

Trajectories	Individuals per trajectory	Average posterior probability for each group	Odds of correct classification	Odds of correct classification (weighted by posterior probability)	Estimated proportion of each trajectory	Observed proportion of each trajectory
Low dose discontinuers	247 (61.70%)	99.20%	78.52	77.53	61.14%	61.44%
Low dose escalators	157	97.97%	75.74	76.70	38.86%	38.56%

### High-dose persisters

**Fig. 4:**
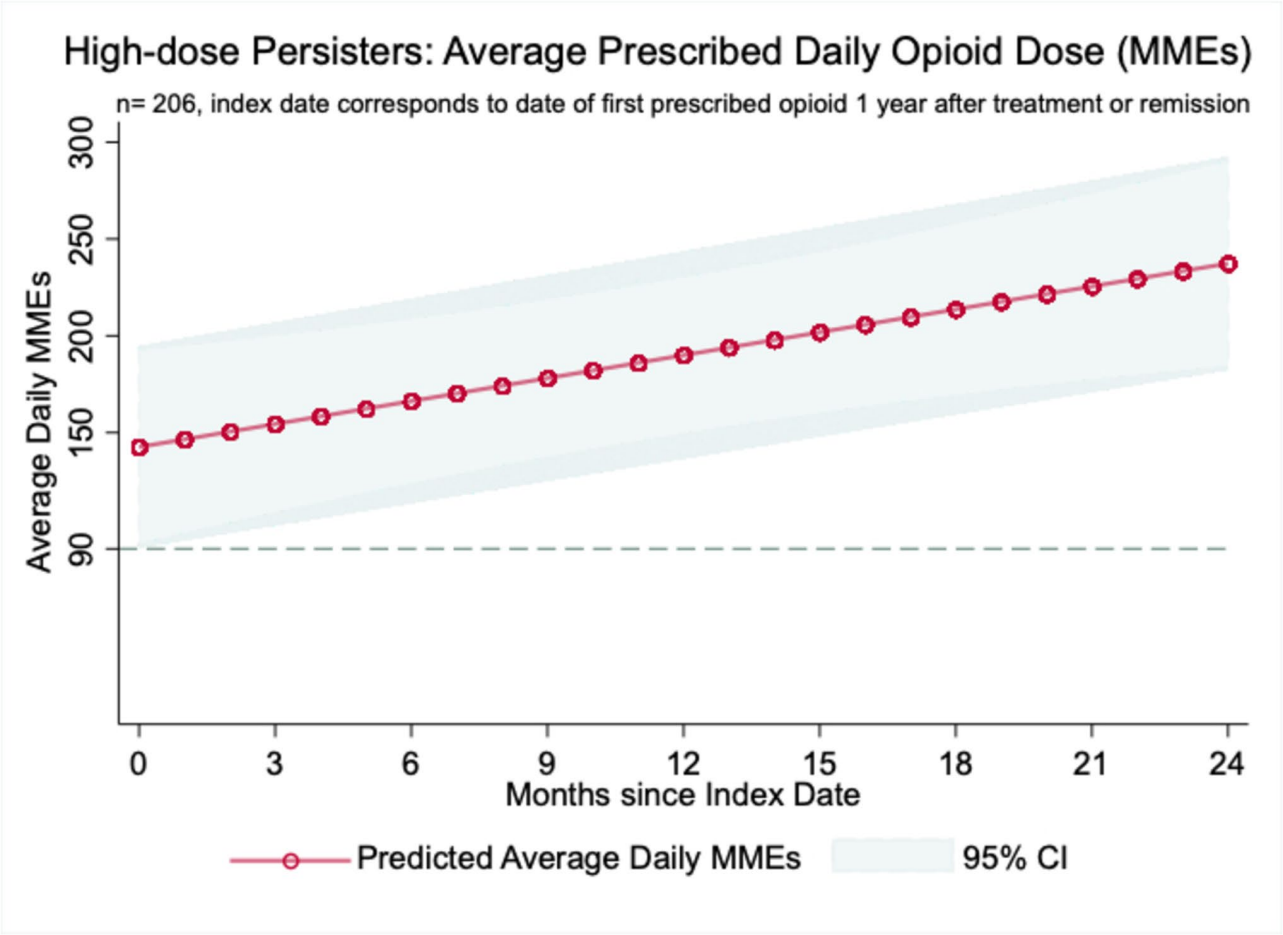
LME model of high-dose persisters

In our preliminary sample, only *n* = 206 patients were found to use opioids at an average daily dose of ≥ 50 MMEs 1 year after completion of their active cancer treatment. Convergence of group-based trajectory models was not possible due to small sample size (*n* = < 300) and widely unbalanced data [37]. Alternatively, linear mixed-effects regression (LME) models were deployed to describe the average daily dose of opioids for 24 months. This alternative approach is useful for unbalanced data, with a differing number of observations per individual over time and permits the inclusion of time-variant and -invariant predictors of the population-level trends [40, 41]. Unlike low-dose persisters, characteristics such as living in a rural zip code, being female, or Black were not found to be statistically significant, when examined in isolation or in an adjusted model. Notably, the predicted results from the LME model (Wald χ^2^ = 8.46, *p*-value = 0.004) show that on average high-dose persisters experienced their opioid dose increase to an average of ~ 250 MMEs/day within 24 months (Fig. 4).

In both models, cancer type, age at diagnosis, or cancer treatment were not statistically significant risk factors.

## Discussion

Opioids are a cornerstone for pain management in cancer patients with moderate to severe pain, especially at the time of cancer diagnosis and throughout active cancer treatment. However, there are limited studies describing patterns of opioid utilization years after completion of cancer treatment with curative intent. This study describes the trajectories of opioid prescriptions in disease-free CS at a single NCI-designated comprehensive cancer center who are at least 1 year beyond completion of their cancer treatment. Importantly, the focus of this study resides on the dose exposure to opioids over time as opposed to OUD-related harms or outcomes. While prior research has examined the patterns of opioid use within the first year of cancer diagnosis, this is the first study examining the trajectories of opioid dose over time in disease-free cancer survivors who are at least one or more years beyond completion of their active cancer treatment [4, 29, 44]. This is important as this study examines opioid dose over time in disease-free CS who are, therefore, thought to be without ongoing tissue damage from cancer or treatment-related effects [5, 45]. In the non-cancer population, numerous studies point to the limited or lack of benefit of opioids in treating chronic pain, with multimodal pain management strategies being more effective [46–48]. Although disease-free CS can experience ongoing pain such as neuropathy and plexopathy from cancer treatments, the role of opioids in chronic pain management, especially high-dose opioids, is unclear [46, 49].

In our trajectory analysis of prescription patterns, more than a third (36.1%) of disease-free CS had increasing doses of up to 100 MMEs/day 3 years after completion of treatment. Our findings are consistent with previous larger population-level studies that quantified the prevalence of chronic pain and overall opioid use in cancer survivors. Jiang and colleagues have shown that 36.4% of patients with a history of cancer report chronic pain, while Lea and colleagues demonstrated how all cancer survivors 1 year after surgery with curative intent were still filling high-dose opioid prescriptions (≥ 90 MMEs/day) [2, 50]. In addition to management of chronic pain during disease-free survivorship, we cannot ignore the possibility of opioid-induced tolerance to explain the dose-escalating trajectories observed in the results. The patients included in this study have been exposed to opioids during the treatment and, at least, 1 year after conclusion of treatment. During this prolonged time, it is possible that continued exposure to opioids resulted in a progressive decrease in analgesia, as described by Mercadante and colleagues [51]. Continued escalating doses of opioids have also been described as potential risk factors for opioid-induced hyperalgesia (OIH), in which nociceptive sensitization is heightened [52, 53]. Patients with OIH report stronger perceptions of pain, inducing prescribers to increase doses [54]. Tolerance is a known dimension of dependence and addiction risks [52, 53]. Importantly, the data source used in this study did not allow determination of the reasons for opioid dose escalation, rendering the abovementioned reasons as conjectures. Therefore, it is important to determine whether high-dose opioid exposure during long-term survivorship is associated with adverse opioid- and cancer-related outcomes, and the causes for escalating opioid doses. Additionally, it is important to assess the impact of health disparities on opioid-related outcomes [52].

Additionally, the trajectory analysis describing the prescription patterns of opioids in low-dose persistent users (< 50 MMEs/day at the index date) shows that more than a third (38.3%) of these patients continue using increasing doses of opioids up to 100 MMEs/day 3 years after completion of treatment or remission. Surprisingly, type of cancer was not associated with opioid escalation trajectory membership. In this sample, the types of cancer are somewhat distributed, with only two meaningful groups: gynecologic/breast cancers and leukemia/lymphoma. Therefore, it is possible that the logistic model approach may not find enough statistically meaningful differences between discontinuers and escalators.

When both low- and high-dose groups were combined, 59.18% of disease-free CS exhibited increasing doses of opioids (i.e., *n* = 155 from the low-dose escalators and *n* = 206 from high-dose persisters). While we do not claim our cohort is representative of the national cancer survivor population, the shape of the trajectory of the low-dose persisters is informative for a number of reasons. First, it highlights the rate of increase in dose for patients using less than 50 MMEs/day 1 year after completion of cancer treatment. In this group, the benefit of managing pain with increasing doses of opioids in disease-free cancer patients who are not undergoing active cancer treatment or have experienced cancer recurrence is unclear. The same conclusion can be made for the patients taking $\ge$ 50 MMEs/day. On average, high-dose persisters are taking approximately 150 MMEs/day 1 year after conclusion of treatment and their average daily dose increases to almost 250 MMEs/day within 2 years. Although a comprehensive evaluation for the reasons behind dose escalation was beyond the scope of this project, future studies examining both patient and provider factors for continued opioid use and dose escalation are warranted.

While we recognize the limitations of our single-center study, our findings still provide valuable insights into how disease-free CS continue to be exposed to opioids, and in many cases, escalating doses, years after treatment conclusion. By examining the trajectory of opioid use in disease-free CS who are at least 1 year out from active cancer treatment, this study sheds light on issues that warrant further investigation, particularly the provider and patient factors associated with ongoing use of opioids and the need for multidisciplinary chronic pain management in this population. Furthermore, the findings of this study were obtained using data spanning 20 years, which were characterized by several significant changes in the public health responses to the opioid epidemic in the USA, including constant updates to the clinical guidelines, and the prescription and dispensing regulatory framework. All of these combined could influence the findings of this study. However, this study provides an innovative insight into how to characterize the extent (i.e., dosing trajectories) to which cancer survivors are exposed to opioids and for how long, after conclusion of treatment. Opioids clearly have a role in treating moderate to severe acute cancer pain, but the best approach to chronic pain management is multidisciplinary pain management and not escalating doses of narcotics [55, 56]. This raises additional questions about whether opioid-related harms in CS have been sufficiently evaluated [25, 29, 57, 58]. Most of the published studies suggest cancer survivors are not at higher risk for opioid-related harm compared to the general population, but this evidence is largely comprised of studies limited to the perioperative period [44, 59, 60]. Moreover, opioid-related harm is recorded by specific ICD-10 diagnoses, which may be underreported due to factors such as underdiagnosis from lack of provider training and the stigma related to OUD [61–63].

Given the increasing numbers of CS and the growing numbers of long-term CS, future studies should examine risks, benefits, and indications for ongoing opioid prescription in disease-free CS as well as the role of early multidisciplinary chronic pain management in providing comprehensive care for all cancer survivors.

## Conclusion

This study provided insights into the long-term patterns of opioid use among disease-free cancer survivors, highlighting a significant concern regarding persistent and increasing opioid use years after completion of cancer treatment. These findings suggest that a proportion of disease-free cancer survivors on chronic opioids require increasing doses over time. This raises important questions regarding the use of opioids for chronic pain management in disease-free cancer survivors and the associated harms of opioid use, including risks of developing opioid use disorder (OUD). The study's trajectory analysis of low-dose and high-dose persistent users underscores the need for ongoing monitoring and tailored interventions to address the unique pain management needs of cancer survivors while mitigating the risks of prolonged opioid use.

## Data Availability

No datasets were generated or analysed during the current study.
